# Working Men and Hospital Legacies

**Published:** 1914-03-07

**Authors:** 


### WORKING MEN AND HOSPITAL LEGACIES.

Some of the most successfully administered voluntary hospitals in the provinces, for the conditions are not at all the same in London, are those which rely on the keen interest and generous contributions of a large number of small subscribers, of whom the working men, through their associations, are the chief. This fact influences policy in more ways than one. Not only does it raise the question of representation, as we saw, for instance, at the annual meeting of Bristol Royal Infirmary last week, but it also provides considerations for the spending of legacies which the hospital receives.

For instance, at a meeting of the governors of King Edward VII.'s Hospital at Cardiff, which we report elsewhere in this issue, General Lee, the Chairman of the Board, urges that certain funds, amounting to some £5,000, which were bequeathed or given for the general purposes of the institution, should not be invested, but used to reduce the overdraft on the buildings account. He based his argument on the facts that the invested funds of the hospital already amounted to some £130,000, and that the overdraft on the working account had practically disappeared. He ended with the plea that if his suggestion was followed the remaining unoccupied fifty beds could be opened, and thus help to reduce the waiting list of 800, which has become chronic. General Lee is perfectly correct in the policy he advocated, which can be safely followed at Cardiff. Conservative finance requires that a voluntary hospital shall have free money invested as a minimum reserve to an amount equal to five times its average ordinary expenditure. Cardiff has at present invested funds which exceed this minimum by £44,000. General Lee's proposal is one, therefore, which may safely be carried out, and it should win the support of the working classes as good business and because the fifty additional beds are urgently needed at Cardiff.

It may be laid down as a sound principle that there is a limit to the amount of invested funds which a voluntary hospital ought to maintain. A completely endowed hospital is not to be worked for. Such an institution forfeits the corporate interest of large bodies of men whose disinterested activities otherwise provide its income, and who thereby become personally bound up with its progressive efficiency, workers for its improvement, trustees of its reputation, and themselves the gainers by the spirit of personal service which the hospital brings into their lives. Most voluntary hospital authorities, however, realise that complete endowment is not a danger which they have any need to fear. But we could point to some institutions where a large capital had already proved a snare and a disaster, till retribution came in the form of setting them to work to regain what they had unwisely mismanaged. Let testators, then, indicate in their wills, if they are anxious about their legacies, that these should be invested to perpetuate their annual subscriptions, and their peace of mind and that of the working men will be secured. No rule can be laid down in the matter, but while pressure from the working men in favour of investment is, generally speaking, an influence for good, they, too, must remember that there is a time to invest and a time to refrain from investing, arguments for the latter certainly being the absence of an overdraft on the working account, a heavy waiting list, and a number of empty beds ready to be opened. The question at Cardiff is complicated by the fact that it is naturally wished to have all the hospital's beds occupied now that the new medical school is coming into existence, and this fact probably will have as much force as any in deciding how the legacies mentioned above shall be employed.

